# Micafungin effect on *Pseudomonas aeruginosa* metabolome, virulence and biofilm: potential quorum sensing inhibitor

**DOI:** 10.1186/s13568-023-01523-0

**Published:** 2023-02-20

**Authors:** Duaa M. Hijazi, Lina A. Dahabiyeh, Salah Abdelrazig, Dana A. Alqudah, Amal G. Al-Bakri

**Affiliations:** 1 Department of Pharmaceutical Sciences, School of Pharmacy, The University of Jordan, Amman, 11942 Jordan; 2 Department of Pharmaceutical Chemistry, Faculty of Pharmacy, University of Khartoum, 1996, 11115 Khartoum, Sudan; 3 Centre for Analytical Bioscience, Advanced Materials and Healthcare Technologies Division, School of Pharmacy, University of Nottingham, Nottingham, NG7 2RD UK; 4 Cell Therapy Center, The University of Jordan, Amman, 11942 Jordan; 5 Department of Pharmaceutics and Pharmaceutical Technology, School of Pharmacy, The University of Jordan, Amman, 11942 Jordan

**Keywords:** *Pseudomonas aeruginosa*, Quorum sensing, Virulence factors, Biofilm, Micafungin, Metabolomics

## Abstract

The prevalence of antibiotic resistance in *Pseudomonas aeruginosa* places a heavy burden on the health care sectors urging the need to find alternative, non-antibiotic strategies. The interference with the *P. aeruginosa* quorum sensing (QS) system represents a promising alternative strategy to attenuate the bacterial virulency and its ability to form biofilms. Micafungin has been reported to impede the pseudomonal biofilm formation. However, the influences of micafungin on the biochemical composition and metabolites levels of *P. aeruginosa* have not been explored. In this study, the effect of micafungin (100 µg/mL) on the virulence factors, QS signal molecules and the metabolome of *P. aeruginosa* was studied using exofactor assay and mass spectrometry-based metabolomics approaches. Furthermore, confocal laser scanning microscopy (CLSM) using the fluorescent dyes ConA-FITC and SYPRO® Ruby was used to visualize micafungin disturbing effects on the pseudomonal glycocalyx and protein biofilm-constituents, respectively. Our findings showed that micafungin significantly decreased the production of various QS-controlled virulence factors (pyocyanin, pyoverdine, pyochelin and rhamnolipid), along with a dysregulation in the level of various metabolites involved in QS system, lysine degradation, tryptophan biosynthesis, TCA cycle, and biotin metabolism. In addition, the CLSM examination showed an altered matrix distribution. The presented findings highlight the promising role of micafungin as a potential quorum sensing inhibitor (QSI) and anti-biofilm agent to attenuate *P. aeruginosa* pathogenicity. In addition, they point to the promising role of metabolomics study in investigating the altered biochemical pathways in *P. aeruginosa*.

**Supplementary Information:**

The online version contains supplementary material available at 10.1186/s13568-023-01523-0.

## Key points

- Micafungin can be used against *P. aeruginosa*.
- Metabolomics is a new approach to study QSI.

**Supplementary Information:**

The online version contains supplementary material available at 10.1186/s13568-023-01523-0.

## Introduction

*Pseudomonas aeruginosa* (*P. aeruginosa*) is an opportunistic Gram-negative bacterium and accounts for many chronic and acute infections (Moradali et al. 2017). It expresses its virulence behavior through a complex network of regulatory circuits known as quorum sensing (QS) (Jimenez et al. 2012; Lee and Zhang 2015). QS is a chemical pathway by which bacterial cells use self-produced signaling molecules, known as autoinducers (AIs), to communicate with each other, and modulate their communal behaviors in a cell-density-dependent manner (Lee and Zhang 2015).

*P. aeruginosa* has three interconnected QS systems, the Las, Rhl, and Pqs, which are mediated by AI signaling molecules (Williams and Camara 2009). Both Las and Rhl are mediated by acyl-homoserine lactone molecules (AHL), while the Pqs is mediated by 2-heptyl-3-hydroxy-4-quinolone (Abisado et al. 2018; Heeb et al. 2011). QS circuits are responsible for the production of several virulence factor such as the phenazine pyocyanin, the siderophore pyoverdine and the glycolipid rhamnolipids (Cornelis and Dingemans 2013; Lee and Zhang 2015; Papenfort and Bassler 2016; Rutherford and Bassler 2012). In addition, QS and its QS-controlled secondary metabolites play an essential role in the ability of *P. aeruginosa* to undergo a distinctive transition from the acute free-living virulent pathogen to the chronic biofilm-adapted pathogen, allowing it to persist for years or even decades (de Kievit 2009; Kostylev et al. 2019). Biofilm is a sessile society of slow-growing cells sequestered within a self-produced extracellular polymeric substance (EPS) (Flemming and Wingender 2010). Matrix EPS in *P. aeruginosa* is composed of various sticky polysaccharides, structural proteins, extracellular nucleic acids (eDNA), and lipids (Di Martino 2018). Biofilm societies exhibit an outstanding resistance toward antibiotics and against the host immune system putting a remarkable challenge to human health (Rasamiravaka et al. 2015). Moreover, the abuse and unwarranted use of antibiotics had accelerated the prevalence of infections caused by multidrug-resistant *P. aeruginosa* strains, leading to the failure of the traditional antibiotic treatment (Pachori et al. 2019). Therefore, an alternative strategy to overcome the rapid and progressive emergence of pseudomonal antibiotics' resistance is urgently needed.

Quorum sensing inhibitors (QSIs) represent a promising strategy to disarm the *P. aeruginosa* from their virulence weapons and to prevent its biofilm full maturation, possibly by reducing the matrix EPS production (Hurley et al. 2012; Srinivasan et al. 2021). Various natural and chemical compounds have been studied as promising QSIs including azithromycin (Tateda et al. 2001), hordenine (Zhou et al. 2018) and resveratrol (Chen et al. 2017). Among these, azithromycin has demonstrated an extensive QS antagonistic activity (Nalca et al. 2006; Tateda et al. 2001) and thus has been used as a standard control in the current investigation. Micafungin, a semi-synthetic antifungal lipopeptide, was reported to impede the pseudomonal biofilm formation by decreasing the transcriptional level of the biofilm pellicles, alginate and 1, 3-β-D-glucan encoding genes (Bazzi et al. 2013; Kissoyan et al. 2016; Rasheed et al. 2018). Nevertheless, the exact mechanism by which micafungin exerts its antibiofilm activity is not fully understood, and literature on its effect on the pseudomonal QS and QS-controlled virulence factors is lacking.

Metabolomics is defined as the qualitative and quantitative analyses of the whole set of metabolites (e.g., amino acids, carbohydrates, lipids, peptides, and nucleotides) within a biological sample (Zhou et al. 2012). These metabolites are firmly related to the phenotypic and the physiologic state of living organisms (Fiehn 2002; Guijas et al. 2018). Hence, tracking the changes in their levels as a result of a disease or a drug intake would afford insight into the mechanisms underlying a pathophysiological illness or a drug’s activity (Everett 2015; Wishart 2016; Zhan et al. 2018). Nowadays, liquid chromatography-mass spectrometry (LC–MS)-based metabolomics is considered as a highly sensitive and comprehensive analytical approach that is widely used in identifying potential biomarkers that can be used to monitor the efficacy of drug therapies (Zhang et al. 2020). Metabolomics was used to investigate the underlying mechanism of various QSIs such as azithromycin (Chen et al. 2017; Phelan et al. 2015), and to effectively differentiate various *P. aeruginosa* strains (Borgos et al. 2015).

Besides metabolic profiling, microscopical examination of microbial biofilms is a promising tool to make a morphological assessment of the effect of various novel strategies on the biofilm appearance and on the spatial architecture of the biofilm intimate details components (Relucenti et al. 2021). Confocal laser scanning microscopy (CLSM) is the technique of choice to inspect the live bacterial biofilms (Schlafer and Meyer 2017), and to image the most detailed ultrastructural components of the biofilm using specialized fluorescent stains (Birarda et al. 2019). In the current study, the effect of micafungin on the virulence factors, QS signal molecules and the metabolome of *P. aeruginosa* was studied using exofactor assay and LC–MS-based metabolomics approaches. Furthermore, CLSM using the fluorescent dyes ConA-FITC and SYPRO® Ruby was used to visualize micafungin disturbing effects on the pseudomonal glycocalyx and protein biofilm-constituents, respectively.

## Materials and methods

### Reference standards, chemicals and reagents

Ammonium carbonate and micafungin were purchased from Sigma-Aldrich (Gillingham, UK), whereas azithromycin dihydrate was kindly donated by Al-Hikma Pharmaceutics (Amman, Jordan). The rest of the reference standards and chemicals were purchased, unless otherwise stated, from either Sigma-Aldrich (Gillingham, UK) or Fisher Scientific (Loughborough, UK). Five different analytical standard mixtures containing 235 compounds were prepared at 20 µM concentration in 1:1 methanol:water and used for the identification of metabolites in this study using LC–MS and/or LC–MS/MS. A compiled list of the content and the description of these standards are available elsewhere (Abdelrazig et al. 2020). Acetonitrile (LC–MS grade) was obtained from VWR (West Sussex, UK), water (LC–MS grade) was from Merck (Darmstadt, Germany) whereas methanol (LC–MS grade) and DMSO (HPLC grade) were from Fisher Scientific (Loughborough, UK). Deionized water was prepared using a water purification system (Milli-Q, Millipore, MA, USA).

### Bacterial and fungal culture conditions

*P. aeruginosa* (ATCC® 47085™) and *Candida albicans* (*C. albicans*) (ATCC® 10231™) were obtained from the American Type Culture Collection. (Manassas, Virginia, USA).

*P. aeruginosa* and *C. albicans* stocks were preserved at − 20 °C in nutrient broth (NB) (Oxoid, Hampshire, UK) supplemented with 10% (*v/v* glycerol:NB). For the study, *P. aeruginosa* and *C. albicans* were incubated in NB at 37 °C with a moderate orbital shaking at 100 rpm for 24, 48 h, respectively. The incubated broth cultures of *P. aeruginosa* and *C. albicans* were streaked on nutrient agar plates (Oxoid, Hampshire, UK), incubated statically at 37 °C for 24 h and 48 h, respectively. Plates were preserved in the fridge (4 °C) to be used in a short time.

### Preparation of micafungin and azithromycin stock solution

A Stock solution (2 mg/mL) of micafungin was prepared in autoclaved and pre-filtered distilled water under aseptic conditions. Azithromycin dihydrate stock solution of 2 mg/mL was prepared in DMSO and then sterilized by filtration using 0.22 µm FilterBio® nylon syringe filter (Filter-bio, Nantong City, Jiangsu P.R, China).

### The minimum inhibitory concentration (MIC) of azithromycin dihydrate and micafungin on the growth of *P. aeruginosa* and *C. albicans*

Clinical and Laboratory Standards Institute microdilution guidelines (CLSI) were used to determine the MIC values of azithromycin dihydrate and micafungin against *P. aeruginosa* (CLSI 2015) and to determine the MIC value of micafungin against *C. albicans* (CLSI 2008). The MIC values (n = 3) of azithromycin and micafungin were considered as the lowest concentration which completely inhibited the visible bacterial or fungal growth.

### Evaluating the effects of micafungin and azithromycin on *P. aeruginosa* growth and pyoverdine level

#### Preparation of working bacterial culture

An overnight culture of *P. aeruginosa* in minimal media (prepared as described by Pamp and Tolker-Nielsen (Pamp and Tolker-Nielsen 2007) using glucose as the sole carbon source (MM)) was adjusted using MM to match 0.5 McFarland’s standard *(ca.* 1 × 10^8^ CFU/mL).

#### Biofilm formation

Biofilms of *P. aeruginosa* were allowed to form on glass coverslips for 24 h as previously described by Birarda et al., (Birarda et al. 2019), with some modifications. Briefly, the backside of glass coverslips 22 × 22 mm was covered with Kapton tape to label one side and minimize the biofilm formation on the other side. The sterilized coverslips were inserted into the wells of a 6-well plate. A volume of 40 µL of the prepared 0.5 McFarland standard suspension of *P. aeruginosa* (*ca*. 1 × 10^8^ CFU/mL) was then added into each well of the 6-well plate to obtain a bacterial density of *ca.* 1 × 10^6^ CFU/mL in a final volume of 4 mL MM. The Culture in each well was challenged with either micafungin 100 µg/mL or azithromycin 8 µg/mL, along with a growth control with no treatment and the plate was incubated at 37 °C for 24 h under moderate shaking (100 rpm) using Dk-SI010 incubator (Daiki Scientific co, Seoul, Korea). Following incubation, the effects of the tested drugs on the growth, pyoverdine levels and metabolic profiles of *P. aeruginosa* were investigated using the effluent supernatant. Moreover, the coverslips were used to visualize the formed biofilms using CLSM.

### The effect of micafungin and different azithromycin concentrations on *P. aeruginosa* growth and pyoverdine production level

*P. aeruginosa* biofilms were allowed to grow for 24 h at 37 °C as previously described. Bacterial culture in each well was challenged with a 100 µg/mL micafungin (n = 6), sub-inhibitory concentrations of azithromycin (2, 4, 8, 16, 32 µg/mL) (n = 3 of each concentration), along with a positive control with no treatment (n = 3). At the end of the incubation period, the optical density at 625 nm (OD_625_) of effluent supernatant was measured using Multiskan GO microplate reader (ThermoFisher Scientific, Basingstoke, UK) to determine the bacterial growth. The pyoverdine level in the effluent supernatant was determined spectrophotometrically at 400 nm as previously described (Musthafa and Voravuthikunchai 2015) and the concentration in µM was determined using a calibration curve. The concentration of azithromycin which significantly (*p*-value < 0.05) reduced the level of pyoverdine with no significant change in the bacterial OD_625_ when compared to the positive control, was considered as the QSI concentration, and was used for further investigations.

### Metabolic profiling of the effluent of the pseudomonal biofilm using LC–MS

#### Sample preparation and metabolite extraction

Biofilms of *P. aeruginosa* were developed on glass coverslips in fresh MM supplemented with micafungin (100 µg/mL, n = 12) and azithromycin at a sub-inhibitory concentration (8 µg/mL, n = 12). Untreated biofilms were also grown and served as controls (n = 12). At the end of the incubation period, the supernatant medium effluent of the formed biofilms with and without treatment was diluted eight folds with water. The diluted samples were centrifuged at 13,000 g for 15 min, diluted with cold methanol (kept at − 20 °C for 30 min prior to use) at a ratio of 1:2 (*v/v* sample:methanol), vortexed for 30 s and finally centrifuged at 13,000 g for 5 min. The upper 80% supernatants were dried using vacuum centrifugal evaporator (Concentrator Plus, Eppendorf, Hamburg, Germany) and stored at − 80 °C.

Prior LC–MS, the dried extracts of the supernatant medium effluent (extracellular spent media) of all samples were reconstituted in 90 µL 2:1 methanol: water, vortexed for 60 s, and centrifuged at 13,000 g at 4 °C for 10 min. The upper 60 μL was transferred into HPLC vial with 200 µL glass insert and analysed immediately. A 20 µL from each of the reconstituted extracts were pooled, vortexed and centrifuged at 13,000 g at 4 °C for 5 min and used as a pooled QC sample for the metabolomics analysis. Blanks (n = 3) were prepared using 2:1 methanol:water following the same procedure excluding the extracted samples.

#### Metabolite profiling and identification using high-resolution LC–MS and LC–MS/MS

Dionex U3000 UHPLC system coupled to a tandem quadrupole-Orbitrap mass spectrometer (QExactive-Plus, Thermo Fisher Scientific, Hemel Hempstead, UK) was used for metabolite profiling and metabolite identification. A ZIC-*p*HILIC column (4.6 × 150 mm, 5 μm) (Merck Sequant, Watford, U.K.) maintained at 45 °C and a flow rate of 300 µL/min was used for the chromatographic separation of the extracted samples (10 µL). The mobile phases consisted of 20 mM ammonium carbonate (A) and acetonitrile (B). The separation started with an initial gradient of 20% (A) and increased to 95% (A) over 8 min then returned to 20% (A) in 2 min (at a flow rate of 400 µL/min) before re-equilibrating the column for 5 min at the initial gradient conditions. LC–MS profiling was performed using polarity switching mode of positive and negative electrospray ionization (ESI + , ESI−). The MS operational parameters were kept as follows: spray voltage 4.5 kV (ESI +), 3.5 kV (ESI−), capillary voltage 20 V (ESI +), − 15 V (ESI−), sheath, auxiliary and sweep gas flow rate were: 40, 5 and 1 (arbitrary unit), respectively, for both modes. Capillary and heater temperatures were maintained at 275 °C and 150 °C, respectively. Data was acquired in a full scan mode with resolution 70,000 from mass over charge (*m/z*) 70–1050. Top 10 data-dependent MS/MS (ddMS/MS) accurate mass fragmentation scans were performed on the pooled QC sample (n = 3) for metabolite identification using step-normalized collision energies of 20, 30 and 40 at resolution 17,500.

Blanks (n = 3) and the standard mixtures (n = 5) were analysed followed by the samples (randomized). Pooled QC injections (n = 6) were used to equilibrate the column at the beginning of the sample batch and then they were interspaced throughout the run to check the performance of the analytical system.

#### Data processing, statistical analysis and pathway analysis

Raw LC–MS/MS data of the studied groups (control untreated *P. aeruginosa*, azithromycin treated *P. aeruginosa* and micafungin treated *P. aeruginosa*) was processed with compound Discoverer 3.1 SP1 (Thermo Fisher Scientific, Hemel Hempstead, UK) for untargeted peak-picking, peak alignment, and annotation of related peaks. For multivariate analysis, mass ions (retention time (RT), *m/z*) pairs with their normalized abundances were exported to Simca P + 14 (Sartorius Stedim Data Analytics AB, Umea, Sweden). Imported datasets were mean-centered and Pareto scaled. Principal component analysis (PCA), partial least square discriminative analysis (PLS-DA), and orthogonal PLS-DA (OPLS-DA) were used for modeling the differences between the groups in the study. Cross-validation using the leave-one-out method (1 out of 7) was used to evaluate the robustness of the generated models by monitoring the fitness of model (R^2^X) for PCA, (R^2^Y) for PLS-DA and OPLS-DA and predictive ability (Q^2^) values. Models that yielded large R^2^X, R^2^Y (close to 1), and Q^2^ values of ~ 0.5 indicate a robust model (Worley and Powers 2013). A permutation test (100 permutations) was also performed to validate the OPLS-DA. Mass ions responsible for the class separation were specified using variable importance in the projection (VIP) of the generated OPLS-DA model. A VIP score above 1.0 is considered important for the model (Yu et al. 2021).

For univariate analysis, the processed mass ions with their peak areas were uploaded to MetaboAnalyst version 5.0 (McGill University, Montreal, QC, Canada) (http://www.metaboanalyst.ca) (Pang et al. 2021). The datasets were normalized to the total sample median and Pareto scaled. Unpaired two-tailed Student’s *t*-Test was used to identify significantly altered metabolites between control and each of the two treated groups. False discovery rate (FDR)-corrected* p*-value of less than 0.05 was considered significant. For binary comparisons, a volcano plot was generated applying FDR-corrected *p*-values of less than 0.05 and fold changes (FC) greater than 1.2 (or less than 0.82). Metabolites with FDR < 0.05 and VIP > 1.0 were considered as potential biomarkers and were subjected to pathway analysis using KEGG Database (https://www.genome.jp/kegg/pathway.html) (Mielko et al. 2021).

### Metabolites identification

The identification of metabolite was carried out by matching the accurate masses, the RT and/or ddMS/MS fragmentation pattern of the detected peaks with metabolites in the BioCyc *P. aeruginosa* Database (http://www.biocyc.org), the RT of the reference standards, and the *mz*Cloud fragmentation database, respectively (Thermo Fisher Scientific, Hemel Hempstead, UK). A database of the accurate mass of some expected metabolites was also used to identify these metabolites across the sample datasets. The confidence in metabolite identification was assigned as level 1–4 (L1-L4) based on the recommendation by Chemical Analysis Working Group, Metabolomics Standards Initiative (MSI) (Sumner et al. 2007, 2014). Metabolites with very low confidence in identification were removed and only metabolites with L1 and L2 were reported.

### Biofilm visualization using CLSM

Zeiss confocal LSM 780 microscopy (Zeiss, Oberkochen, Germany) was used to investigate the spatial structural changes in the pseudomonal biofilm-constituents upon treatment with micafungin 100 µg/mL or azithromycin 8 µg/mL using two fluorescent dyes; Concanavalin A FITC conjugate, Type VI (ConA-FITC, Sigma–Aldrich, MO, USA) and SYPRO® Ruby Protein Gel Stain (Sigma–Aldrich, MO, USA) to visualize the matrix polysaccharides and matrix proteins, respectively. On the experiment day, ConA-FITC (50 µg/mL) was prepared with 50 mM phosphate buffer saline (PBS, pH 7.4) supplemented with 10 µL of 1 mM CaCl_2_ and 10 µL of 1 mM MnCl_2_. Formed biofilm was incubated with 350 µL of ConA-FITC for 50 min in darkness at room temperature and then washed with PBS to remove excess stain. The fluorescent biofilm images were then acquired using CLSM oil immersion lens with the emission and excitation wavelength of 520 nm and 488 nm, respectively. For protein visualization, formed biofilm was incubated with 350 µL of SYPRO® Ruby for 60 min at room temperature then observed on CLSM using 63X0.9NA 2.2 mm water immersion objective with the emission and excitation wavelength of 610 nm and 450 nm, respectively.

### Statistical analysis

All experiments were performed in a minimum of three replicates and the results were presented as mean ± standard deviation (SD). Statistical analysis was conducted using GraphPad Prism Software version 8.01 (GraphPad, San Diego, CA, USA). The differences among groups were determined by one-way ANOVA followed by the Dunnett test as appropriate, while independent Student’s *t*-Test was used for binary comparisons, *p*-values < 0.05 were considered statistically significant.

## Results

### Minimum inhibitory concentration (MIC)

The MIC values of micafungin against *P. aeruginosa* and *C. albicans* are presented in Table 1. When tested against *C. albicans*, micafungin had a MIC value of 48.8 ng/mL. Micafungin showed no inhibitory effect on *P. aeruginosa* growth at a concentration of 100 µg/mL and lower. Therefore, micafungin concentrations ≤ 100 µg/mL were considered sub-inhibitory towards *P. aeruginosa* culture. The MIC value of azithromycin against *P. aeruginosa* was 64 µg/mL (Table 1), therefore, azithromycin concentrations < 64 µg/mL were considered sub-inhibitory towards *P. aeruginosa* culture.

**Table 1:** MIC of Azithromycin and Micafungin

Microorganism	Drug	MIC^a^
*P. aeruginosa*	Azithromycin	64 µg/mL
*P. aeruginosa*	Micafungin	> 100 µg/mL
*C. albicans*	Micafungin	48.8 ng/mL

^a^Data presented as the mean of triplicates from three independent experiments

### The effects of micafungin and azithromycin on *P. aeruginosa* growth and pyoverdine levels

**Fig. 1:**
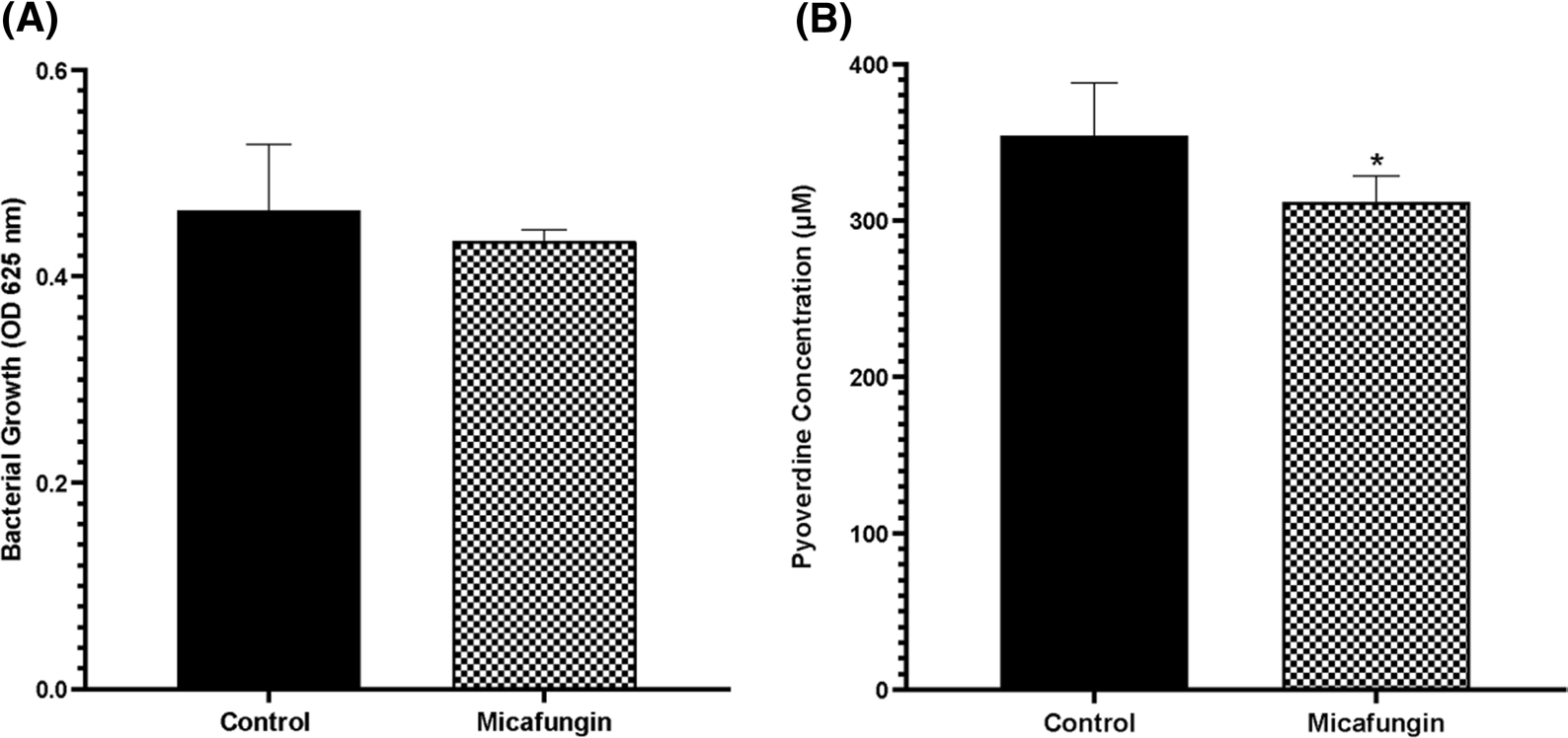
The effect of micafungin 100 µg/mL along with control [without any treatment (n = 6)] on **A** the growth (OD_625_) and **B** pyoverdine production of *P. aeruginosa*. Data was analysed by independent Student’s *t*-test. Significant in comparison to control is expressed as * *P*-value ≤ 0.05

QSI concentration is considered as the concentration which significantly decreases the production of pyoverdine with no significant effect on the bacterial growth and viability (OD_625_) when compared to the positive control. When compared to the positive control, treating *P. aeruginosa* with 100 µg/mL Micafungin did not affect the growth of the bacteria significantly (*P*-value 0.2865) (Fig. 1A), but was associated with a significant decrease (*P*-value 0.0193) in the production level of pyoverdine (Fig. 1B), and thus this concentration was used for further investigations.

**Fig. 2:**
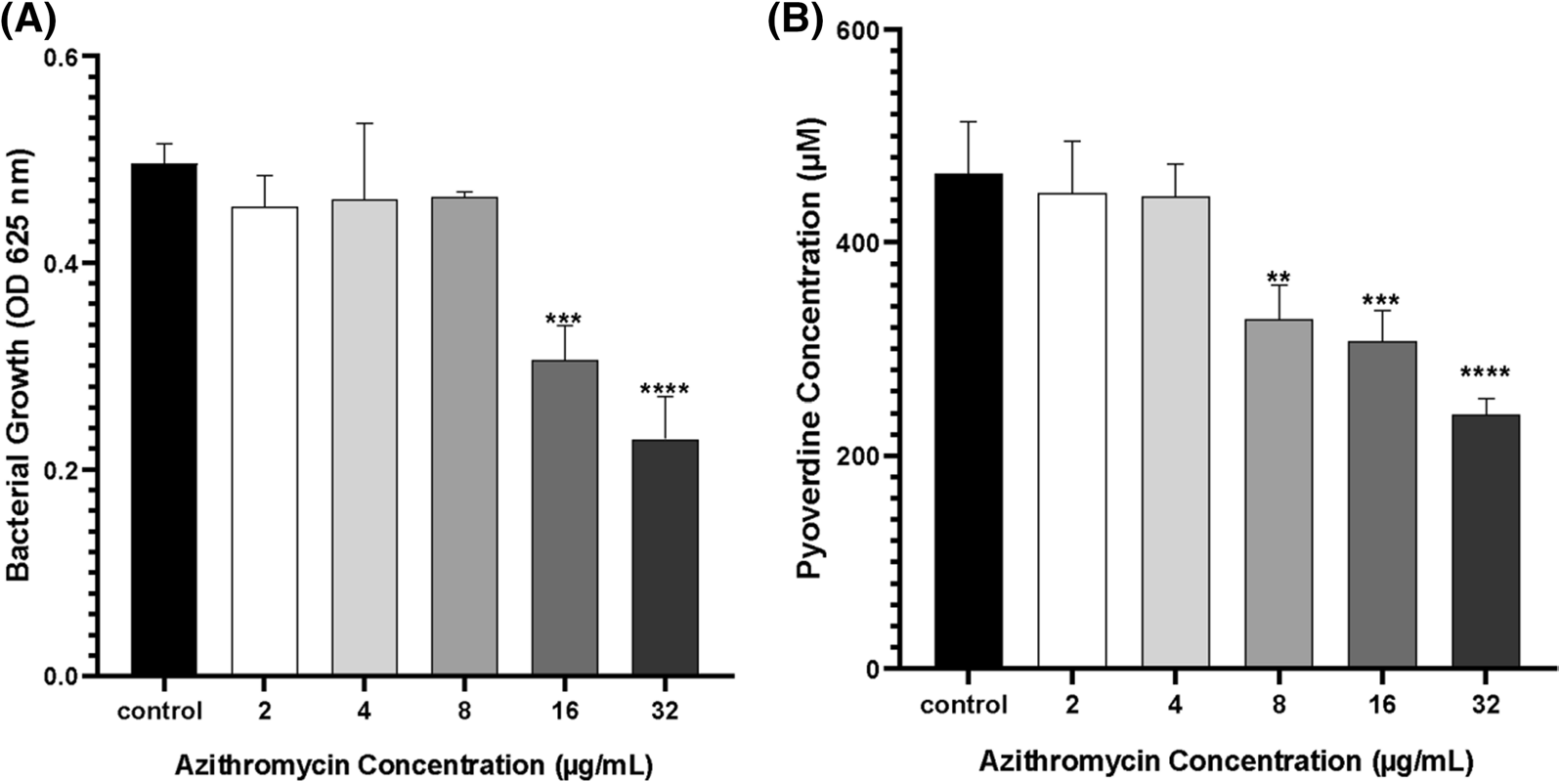
Concentration-dependent effect of azithromycin (0–32 µg/mL) on **A** *P. aeruginosa* growth after 24 h incubation. **B** Level of pyoverdine (µM) production by *P. aeruginosa* after 24 h incubation. Error bars indicate SD from three independent replicates. Data was analysed by one-way ANOVA followed by the Dunnett test as post-hoc. Significance in comparison to control is expressed as ^**^ *P*-value ≤ 0.01, ^***^ *P*-value ≤ 0.001, ^**** ^*P*-value ≤ 0.0001

Different sub-MIC concentrations of azithromycin (2, 4, 8, 16, 32 µg/mL) were tested on *P. aeruginosa* culture to choose an azithromycin QSI concentration to be further tested. (Fig. 2). When compared to control, 16 and 32 µg/mL azithromycin significantly affected the bacterial growth while the remaining concentrations did not (Fig. 2A). Our results showed a concentration-dependent decrease in pyoverdine production upon treatment with increasing concentrations of azithromycin (Fig. 2B). Azithromycin at 8 µg/mL concentration did not significantly affect the bacterial growth (*p*-value of 0.7633) relative to the positive control but was associated with a significant decrease in the level of pyoverdine (*p*-value < 0.01). Therefore, this concentration of azithromycin was considered as the QSI concentration and was adopted for the following experiments.

### Metabolic profiling of the effluent of the pseudomonal biofilm using LC–MS

#### overview

Untargeted LC–MS-based metabolomics was used to investigate the influence of micafungin and azithromycin on the QS signaling molecules, virulence factors and the extracellular metabolome of *P. aeruginosa*. Untargeted LC–MS analysis of the biofilm effluent with and without treatment detected a total of 1202 mass features in both ESI + /ESI− modes. Out of these detected mass features, a total of 125 metabolites were identified.

**Fig. 3:**
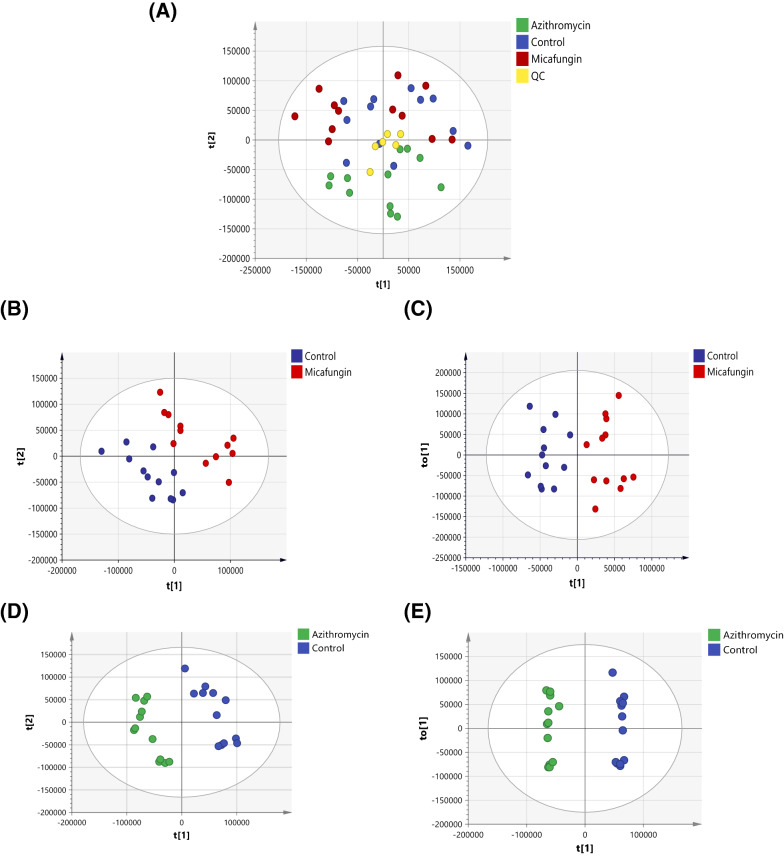
Scores plots of *P. aeruginosa* samples after 24 h treatment with azithromycin (green circles) or micafungin (red circles) along with control without any treatment (blue circles). **A** PCA with quality control samples (QC, yellow points, n = 6), R^2^X = 0.653, Q^2^ = 0.325, n = 12. **B** PLS-DA (R^2^X = 0.531, R^2^Y = 0.985, Q^2^ = 0.746, n = 12) and **C** OPLS-DA scores plots (R^2^X = 0.407, R^2^Y = 0.859, Q^2^ = 0.455, n = 12). **D** PLS-DA (R^2^X = 0.604, R^2^Y = 0.992, Q^2^ = 0.947) (**E**) and OPLS-DA (R^2^X = 0.604, R^2^Y = 0.992, Q^2^ = 0.916)

PCA scores plot (Fig. 3A) of the three groups revealed a good clustering of the pooled QC samples indicating satisfactory stability and a valid analytical performance of the LC–MS. The PCA model showed a partial separation of the azithromycin treated samples from the other two groups. However, no good separation between the untreated control and micafungin treated groups was observed (Fig. 3A).

### Significantly altered metabolites in multivariate analysis

PLS-DA and OPLS-DA models were generated to extract significantly altered metabolites (VIP > 1) responsible for the class separation between micafungin and control samples (Fig. 3B, C). OPLS-DA model showed a better separation over the PLS-DA model, with satisfactory R^2^Y (0.859) and Q^2^ (0.455) values. The OPLS-DA model also passed the validity permutation test (Additional file 1: Fig. S1A). A total of 188 discriminatory mass feature ions (VIP > 1) were responsible for the noticed classes’ separation between micafungin and control. Out of these mass ions, 21 were identified and annotated (Table 2). Among the significantly altered metabolites upon treating *P. aeruginosa* with micafungin are several Las/Rhl signaling molecules (n-tetradecanoyl-l-homoserine lactone, n-3-hydroxyoctanoyl-l-homoserine lactone and n-3-Hydroxytetradecanoyl-l-homoserine), signaling molecules of the Pqs circuits (2-methyl-4-hydroxyquinoline, 2-n-heptyl-4-hydroxyquinoline-n-oxide, 2-heptyl-4-quinolone and 2-monyl-4-hydroxyquinoline n-oxide), several pseudomonal virulence factors (e.g. pyocyanin, rhamnolipid), pyocyanin precursor (phenazine-1-carboxylate) and other metabolites related to specific biochemical pathways such as lysine degradation, valine, leucine and isoleucine biosynthesis, and biotin metabolism (Table 2).

**Table 2:** Significantly altered extracellular metabolites of *P. aeruginosa* biofilm effluent treated with micafungin as extracted from the OPLS-DA scores plot

Putative Metabolite	KEGG ID	Main Pathway^a^	Accurate Mass (Da)	VIP	FC^b^
Phenazine-1-carboxylate	C21442	Phenazine biosynthesis	224.058	8.6	DOWN
8-amino-7-oxononanoate	C01092	Biotin metabolism	187.121	5.8	DOWN
Pyocyanin*	C01748	Phenazine biosynthesis	210.079	3.8	DOWN
(Indole-3-yl) acetate*	C00954	Tryptophan metabolism	175.063	2.4	DOWN
Caprolactam	C06593	Caprolactam degradation	113.084	2.2	DOWN
l-leucine	C00123	Valine, leucine and isoleucine biosynthesis	131.094	2.1	DOWN
Pimelate	C02656	Biotin metabolism	158.058	2.1	DOWN
2-n-heptyl-4-hydroxyquinoline-n-oxide	NA	NA	259.157	2	DOWN
Citraconate	C02226	Valine, leucine and isoleucine biosynthesis	130.027	1.6	DOWN
Pyochelin	C12037	Biosynthesis of siderophore group nonribosomal peptides	324.06	1.6	DOWN
2-methyl-4-hydroxyquinoline	C21873	Biosynthesis of secondary metabolites	159.068	1.5	DOWN
Salicyl alcohol	NA	NA	124.052	1.4	DOWN
Rhamnolipid	NA	NA	650.388	1.4	DOWN
Palmitate	C00249	Fatty acid degradation	256.24	1.4	DOWN
3-acetylamino-4-hydroxybenzoate	NA	NA	195.053	1.3	DOWN
2-Nonyl-4-hydroxyquinoline n-oxide*	NA	NA	287.188	3.4	UP
1-piperideine-2-carboxylate*	C04092	Lysine degradation	127.063	2.6	UP
6-amino-2-oxohexanoate*	C03239	Lysine degradation	145.074	1.8	UP
Catechol*	C00090	Degradation of aromatic compounds	110.037	1.7	UP
Azelaic acid	NA	NA	188.105	1.4	UP
o-acetyl-l-serine†	C00979	Cysteine and methionine metabolism	147.053	1	UP

Metabolites are listed according to their VIP values within the same FC category

*NA* undetected metabolites in KEGG Database

^a^Main pathway as identified by KEGG Database

^b^FC: Fold change. Up and down refer to metabolites up-regulated or down-regulated compared to control (Untreated *P. aeruginosa* culture)

^*^Significant Metabolites identified in both multivariate and univariate analyses

^†^Metabolite identified as Level 1 confidence in identification, the rest of metabolites were identified as Level 2

For azithromycin, a clear separation between the azithromycin and the control group was observed in both PLS-DA (R^2^X = 0.604, R^2^Y = 0.992, Q^2^ = 0.947), and OPLS-DA (R^2^X = 0.604, R^2^Y = 0.992, Q^2^ = 0.916) models with satisfactory scores plot values (Fig. 3D, E). The OPLS-DA model was validated by the permutation test (Additional file 1: Fig. S1B). A total of 169 mass feature had VIP > 1, among which 31 features were annotated. These included pseudomonal virulence factors (e.g., mono-rhamnolipid, rhamnolipid, phenazine-1-carboxamide and phenazine-1-carboxylate), and QS-related metabolites such as 2-methyl-4-hydroxyquinoline, 2-n-heptyl-4-hydroxyquinoline-n-oxide and n-3-hydroxyoctanoyl-l-homoserine lactone.

### Significantly altered metabolites in univariate analysis

**Fig. 4:**
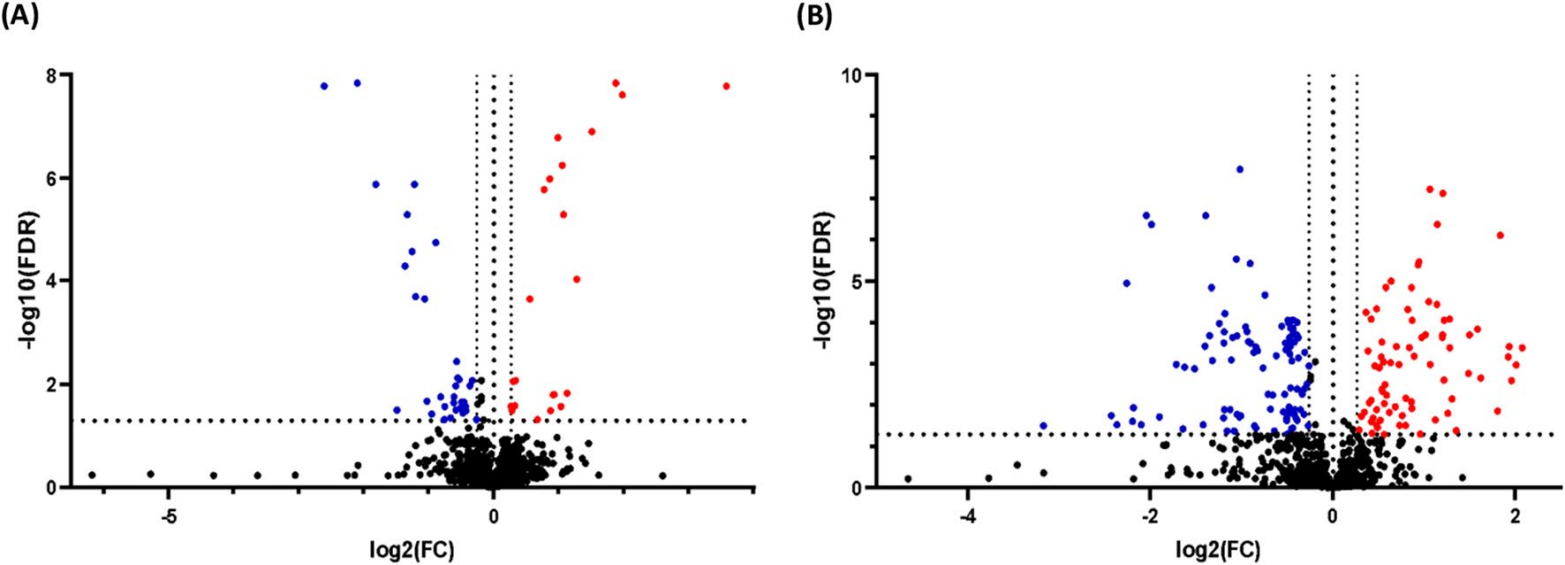
Volcano plot showing the statistically significant up-regulated (red circles) and down-regulated (blue circles) mass features using FDR-corrected *p-*value < 0.05, and FC (greater than 1.2 or less than 0.83) in **A** *P. aeruginosa* treated with 100 µg/mL micafungin and **B** *P. aeruginosa* treated with 8 µg/mL azithromycin, in comparisons to control without treatment

Univariate binary comparison revealed that 61 mass ion features were significantly altered between the micafungin and control classes (FDR < 0.05), eight of which were identified. These eight disturbed metabolites are 1-piperideine-2-carboxylate, catechol, 2-hydroxy-2-methylbutanenitrile, 6-amino-2-oxohexanoate, 2-nonyl-4-hydroxyquinoline n-oxide, (Indol-3-yl) acetate, sulfate and pyocyanin. Binary comparison using volcano plot (FDR < 0.05 value and FC ≥ 1.2 or ≤ 0.83) revealed that treating the developing pseudomonal biofilm with micafungin affected 56 ion masses of which 34 were down-regulated (Fig. 4A).

For azithromycin, a total of 189 mass ions including 40 ions with putative identification were significantly altered (FDR < 0.05). Volcano plot (FDR < 0.05 value and FC ≥ 1.2 or ≤ 0.83) revealed that azithromycin resulted in the perturbation of 181 ions with 102 down- and 79 up-regulated in comparison to control (Fig. 4B). Azithromycin significantly decreased the level of mono-rhamnolipid, 2-nonyl-4-hydroxyquinoline n-oxide, phenazine-1-carboxamide and n-acetyl-l-glutamate 5-semialdehyde. Notably azithromycin was associated with a higher impact on the QS system compared to micafungin as reflected by the larger number of significantly disturbed QS signal molecules.

### Potential biomarker

**Fig. 5:**
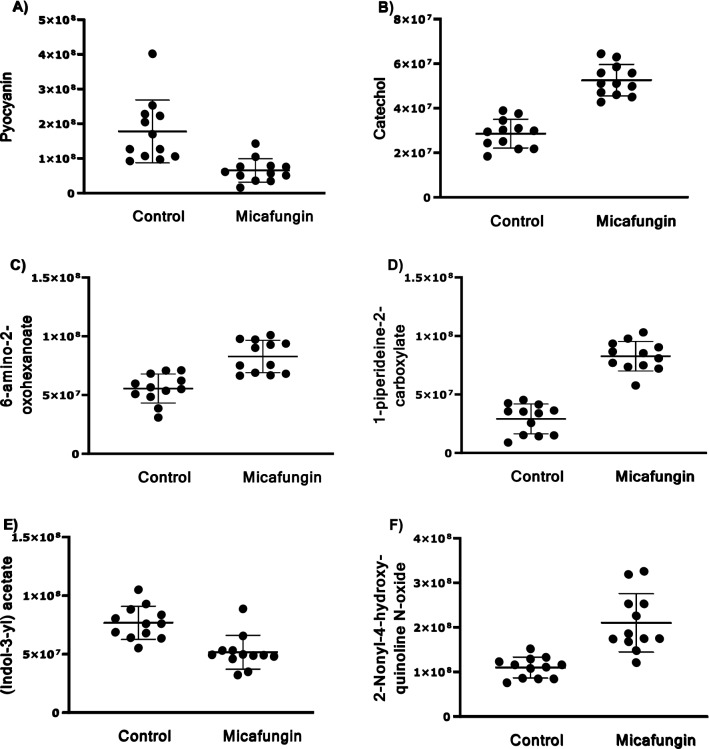
Significantly altered extracellular metabolites (both in univariate and multivariate analysis) in *P. aeruginosa* biofilm effluent treated with micafungin. Data are presented as the peak area for each metabolite as determined by LC–MS/MS analysis in the 12 replicates in control and micafungin groups

Six metabolites were significantly altered in both univariate and multivariate analyses (VIP > 1 and FDR < 0.05) between the control and the micafungin-treated groups (Fig. 5). Pyocyanin and (indol-3-yl) acetate were significantly down-regulated while the level of catechol, 6-amino-2-oxoheanoate, 1-piperideine-2-carboxylate, and 2-Nonyl-4-hydroxy-quinoline N-oxide was significantly increased in the micafungin-treated group compared to controls. These six metabolites are proposed as potential biomarkers and might be used to monitor the effects of micafungin on *P. aeruginosa.* When subjected to pathway analysis, the six dysregulated potential biomarkers were found to be involved in phenazine biosynthesis, tryptophan metabolism, degradation of aromatic compounds, lysine degradation and QS pathways (Table 2).

For azithromycin, 15 metabolites were significantly altered in both univariate and multivariate analyses including the virulence factor mono-rhamnolipid. When subjected to pathway analysis, these metabolites were found to be involved in QS circuits (n-3-hydroxyoctanoyl-l-Homoserine lactone), phenazine biosynthesis (such as phenazine-1-carboxylate), biotin biosynthesis (including 8-amino-7-oxononanoate and pimelate), tryptophan metabolism ((indol-3-yl) acetate), isoleucine biosynthesis (l-leucine and 3-isopropylmalate), and biosynthesis of secondary metabolites (2-methyl-4-hydroxyquinoline). Interestingly, one metabolite (indol-3-yl) acetate was found to be significantly altered with both treatments. However, micafungin significantly decreased (indol-3-yl) acetate level, while azithromycin significantly increased its level and thus further investigation on this metabolite is highly encouraged.

### Biofilm visualization using CLSM

**Fig. 6:**
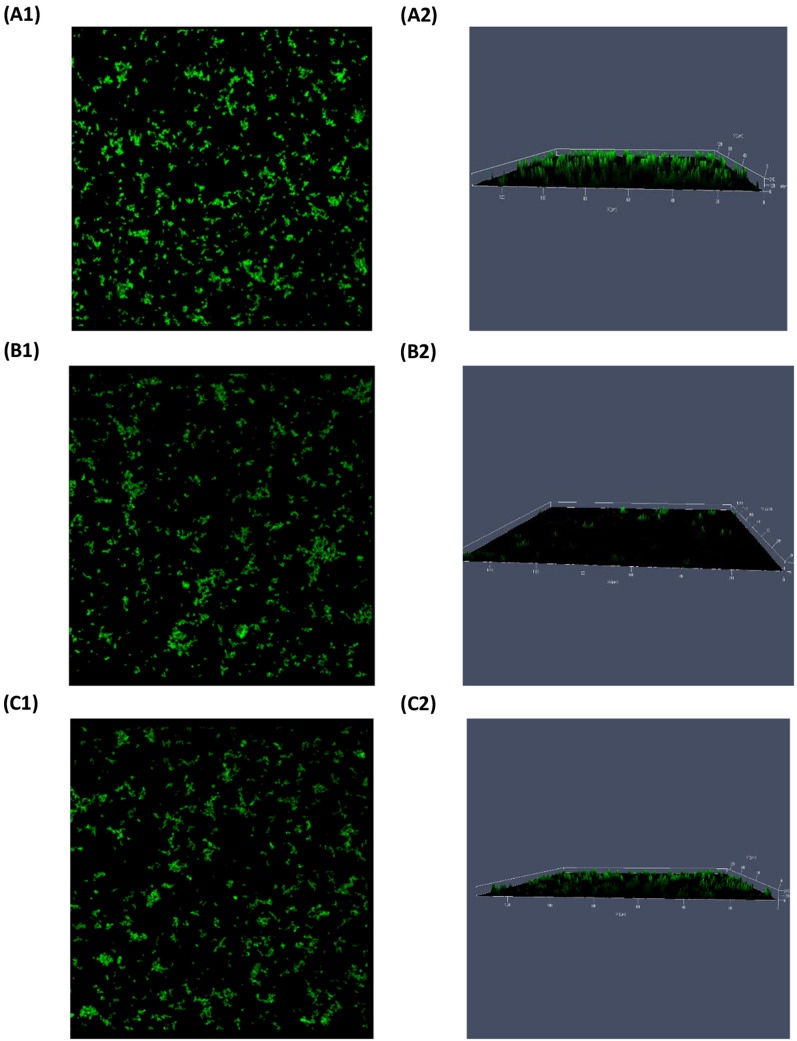
CLSM images of ConA-FITC -stained biofilms obtained after 24 h growth of *P. aeruginosa.* **(1)** refers to the two-dimensional (2D), and **(2)** refers to the 2.5D view of **A** untreated *P. aeruginosa.* **B** micafungin 100 µg/mL treated *P. aeruginosa.* **C** azithromycin 8 µg/mL treated *P. aeruginosa*

**Fig. 7:**
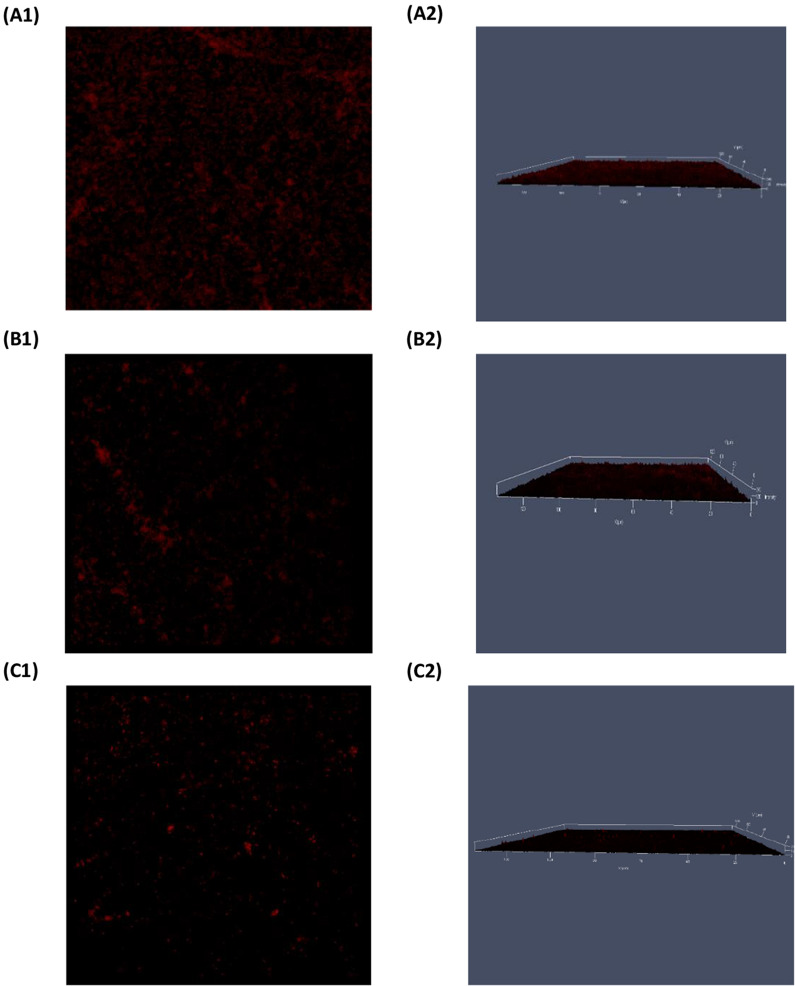
CLSM images of SYPRO® Ruby-stained biofilms obtained after 24 h growth of *P. aeruginosa*. **(1)** refers to 2D, and (**2**) refers to the 2.5D view of **A** untreated *P. aeruginosa.* **B** micafungin 100 µg/mL treated *P. aeruginosa.* **C** azithromycin 8 µg/mL treated *P. aeruginosa*

In the current work, CLSM was used to visualize and explore micafungin disruptive effects on the pseudomonal biofilm-matrix constituents in comparison to the untreated biofilm and beside the biofilms treated with azithromycin 8 µg/mL. ConA-FITC was used to visualize the matrix polysaccharides, while the SYPRO® Ruby was used to insight the matrix glycoproteins. As noted, both treatments induced a disruption in matrix exopolysaccharides (Fig. 6). Moreover, both micafungin and azithromycin obviously disrupted the spatial matrix proteins (Fig. 7).

## Discussion

Currently, the interference with the bacterial QS system represents a promising alternative, and a non-antibiotic strategy to overcome the rapid and progressive emergence of pseudomonal antibiotics' resistance (Kalia and Purohit 2011). In this study, the effects of the antifungal micafungin on the level of *P. aeruginosa* QS regulated extracellular virulence factors, biofilm formation, and on *P. aeruginosa* metabolome, in MM, were investigated for the first time using in vitro exofactor evaluation, CLSM and global MS based-metabolomics approach, respectively. Additionally, the effects of azithromycin (a reported QSI) were also explored as a standard control beside micafungin.

### Effects of micafungin on the pseudomonal QS signaling molecules and QS-controlled virulence factors in the biofilm effluent

Micafungin is an echinocandin antifungal agent which inhibits fungal cell wall synthesis by interfering with beta-(1,3)-d-glucan synthase (Jarvis et al. 2004; Leverger et al. 2019). It has been reported to affect biofilm formation in *P. aeruginosa* (Bazzi et al. 2013; Kissoyan et al. 2016; Rasheed et al. 2018).

LC–MS metabolomics has revealed that treating the growing pseudomonal biofilm with micafungin significantly modulated the dynamics of several metabolic processes. Notably, the multivariate analysis showed a significant dysregulation in the level of several signaling molecules in Las/Rhl and Pqs circuits (Bredenbruch et al. 2005; Ha et al. 2011). Among these signaling molecules the 2-nonyl-4-hydroxyquinoline n-oxide was significantly up-regulated both in univariate and multivariate, and further investigation is intended to thoroughly understand its effects.

**Fig. 8:**
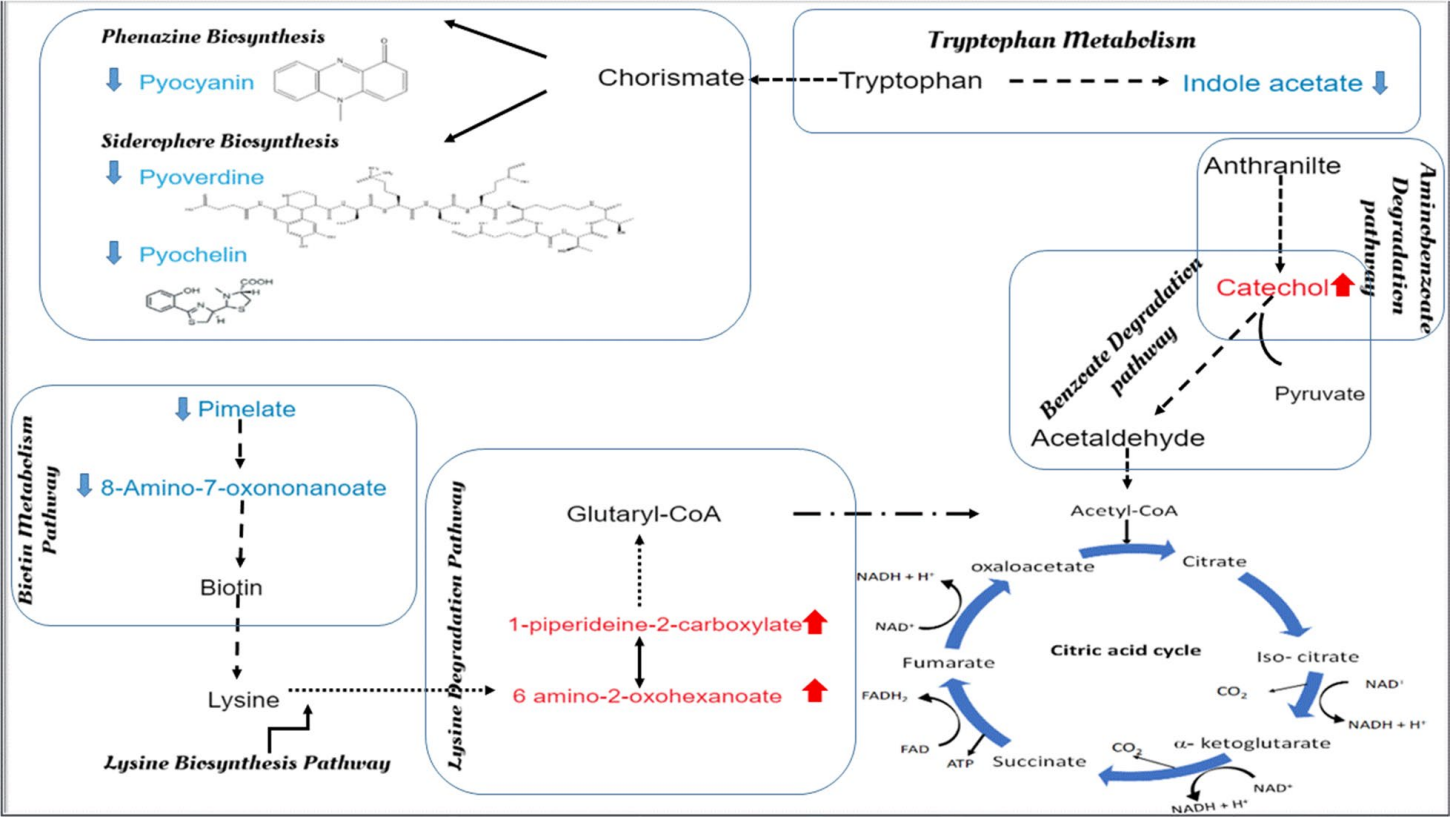
A proposed scheme of the most important altered biochemical pathways of *P. aeruginosa* upon treatment with micafungin. Up-regulated metabolites are shown in red arrow, down-regulated metabolites are shown in blue arrow, dashed arrows indicate multiple enzymatic reaction steps

The effect of micafungin on the QS system is expected to reduce the level of various crucial QS-controlled virulence factors. Herein, micafungin significantly suppressed the level of pyoverdine, pyocyanin and rhamnolipid. Metabolomics study revealed a dysregulation in the siderophore and phenazine biosynthesis pathways involved in pyoverdine and pyocyanin production, respectively. The level of pyochelin, synthesized by siderophore biosynthesis pathway (Stintzi et al. 1998), and pyocyanin and its direct precursor in the phenazine biosynthesis pathway, phenazine carboxylate, (Look et al. 2005), was found to be significantly decreased upon treatment with micafungin (Fig. 8). The latter finding might indicate that micafungin had affected certain enzymatic reactions in the phenazine biosynthetic pathway, which are responsible to produce the phenazine-1-carboxylate from the chorismic acid. Interestingly, metabolomics data revealed a dysregulation in the tryptophan metabolism pathway (Table 2). Tryptophan metabolism is linked to the phenazine and the siderophores biosynthetic pathways which are involved in the production of the two virulence factors, pyocyanin and pyoverdine, respectively (Fig. 8). All will ultimately affect biofilm development, maintenance and infection severity (Banin et al. 2005; Cornelis and Dingemans 2013).

Azithromycin at sub-inhibitory concentrations was reported to possess a QSI effect, inhibit *P. aeruginosa* virulence factor production and affect its biofilm formation (Gillis and Iglewski 2004; Tateda et al. 2001). In the current study, azithromycin demonstrated a concentration-dependent antimicrobial and anti-virulent activity against *P. aeruginosa*. LC–MS metabolomics data showed that azithromycin impaired the level of several QS signaling molecules (e.g., 3-Hydroxy-hexanoyl-l-homoserine lactone) and resulted in a significant reduction in the level of several QS-controlled virulence factors including monorhamnolipid, pyochelin, and the pyocyanin precursor, phenazine-1-carboxylate. These results are consistent with the results reported by Phelan et al. (Phelan et al. 2015). However, in the current work and in contrast to Phelan et al. azithromycin was not associated with a significant reduction in the pyocyanin level (Phelan et al. 2015).

### Effects of micafungin on other extracellular metabolites of *P. aeruginosa*

Beside its effects on the level of QS-virulence factors, micafungin was found to affect other metabolic processes (Table 2) including amino acids biosynthesis (e.g., tryptophan, lysine, valine, leucine and isoleucine), and biotin metabolism. Additionally, treatment with micafungin is proposed to impair the pseudomonal TCA cycle as reflected by the dysregulation in the level of metabolites involved in the synthesis of acetyl CoA and TCA cycle intermediates (Fig. 8).

Catechol, generated from indole acetate in the tryptophan metabolism pathway (Fig. 8), and 1-piperideine-2-carboxylate and 6-amino-2-oxohexanoate, involved in the lysine degradation pathway (Fig. 8), are involved in enzymatic reactions that end up with the production of acetyl CoA. The level of these three metabolites was perturbed in the biofilm effluent of micafungin treated *P. aeruginosa*. The possible impairment in the TCA cycle might ultimately affect the generation of cellular intermediates that serve as biosynthetic precursors for fatty acid, amino acid and carbohydrate, and might also alter energy production pathways (Owen et al. 2002). Although the effect of micafungin on the TCA cycle has not been reported before, it worth mentioning that TCA cycle impairment was not associated with a reduction in the bacterial growth (Fig. 8), and thus further investigation on the TCA is urged. TCA cycle disturbances in *P. aeruginosa* were recently reported with several QSIs including resveratrol, hordenine and methyl-N-methyl anthranilate (Chen et al. 2017; Ma et al. 2021; Zhou et al. 2019). However, their exact mechanism on the suppression of TCA cycle is not fully explained. Nevertheless, as a main source of energy, the impairment in TCA cycle might ultimately lessen *P. aeruginosa* pathogenicity and could restrict its biofilm formation.

Micafungin has altered the biotin metabolism pathway as reflected by the decrease in the level of biotin precursors pimelate and 8-amino-7-oxononanoate (Table 2, Fig. 8). Biotin is a key enzyme cofactor for a range of enzymes (e.g. carboxylases, decarboxylases and transcarboxylases) involved in various biochemical processes including gluconeogenesis, fatty acid biosynthesis and amino acid metabolism (Yuan et al. 2012). Pyruvate carboxylase, responsible for replenishing the TCA cycle with oxaloacetate, is a biotin-dependent enzyme (Zhang et al. 2021). Additionally, biotin is involved in lysine production which will proceed via several enzymatic reactions to result in the production of acetyl CoA (Fig. 8). Taken together, the dysregulation in the biotin metabolism might have played a role in the noted perturbations in the TCA cycle. So far, no study has reported the effect of micafungin on biotin metabolism and TCA cycle pathways. Therefore, further investigations remain needed. It is worth mentioning that most of the current literature on biotin metabolism is based on studies performed on *Escherichia coli* and *Bacillus subtilis* (Zhang et al. 2021). Hence, further studies on *P. aeruginosa* are necessary.

### Biofilm visualization upon treatment with micafungin and azithromycin

CLSM images of micafungin treated biofilm (Fig. 6) demonstrated a level of disruption in matrix-exopolysaccharide when compared to the untreated control. These results are in concordance with the micafungin reported effects on the synthesis of matrix glycocalyx (Psl, Pel and glucan) (Bazzi et al. 2013; Kissoyan et al. 2016; Rasheed et al. 2018). On the other hand, this is the first time to explore micafungin effects on the distribution of the matrix protein as shown in Fig. 7. SYPRO® Ruby images indicated that micafungin could interfere with the protein synthesis. Using CSLM Z-stacking, micafungin (at 10 mg/mL) was reported to decrease pseudomonal biofilm thickness after treatment for two days (Rasheed et al. 2018). However, this is the first study to investigate the spatial and structural changes in the distribution of the biofilm-matrix polysaccharides and proteins upon treatment with micafungin using CLSM and protein and glycocalyx specific fluorescence dyes.

The effect of azithromycin on *P. aeruginosa* biofilm was previously studied using the green fluorescent protein and the Live/Dead stain fluorophores where azithromycin (2 µg/mL) resulted in a minimal biofilm formation after 24 h treatment (Gillis and Iglewski 2004). Herein, azithromycin had a noticeable effect on matrix exopolysaccharides and proteins using ConA-FITC and SYPRO®, however, its effect on the matrix-proteins was more obvious than on the matrix-glycocalyx.

Beside imaging, LC–MS/MS global metabolomics has revealed a significant reduction in the level of several amino acids in the biofilm effluent which might further illustrate and strengthen the results of CLSM.

In conclusion, we studied, the effect of micafungin as a potential QSI against *P. aeruginosa* batch culture for the first-time using MS-based global metabolomics and CLSM approaches. Micafungin had shown a promising anti-pathogenic effect by significantly decreasing the level of various QS-controlled virulence factors including pyocyanin and its direct precursor, pyoverdine, pyochelin and rhamnolipid. Moreover, micafungin had shown a dysregulation in various metabolic pathways including lysine, tryptophan, and biotin, which might propose an impairment activity in the TCA cycle. The reported QSI azithromycin was associated with a higher impact on the QS system compared to micafungin as reflected by the larger number of significantly disturbed QS signal molecules. Nevertheless, the findings presented herein clearly indicate that micafungin has a potential activity on the QS circuits. Our work revealed that micafungin had a promising anti-biofilm property as revealed by the distributional change of matrix-proteins and matrix-exopolysaccharide. This activity might improve antibiotics permeability by decreasing the matrix hindering effects and could be further studied by combining micafungin with antibiotics.

Taken together, the current work highlighted the promising role of micafungin (100 µg/mL) as a potential QSI with promising anti-virulence and anti-biofilm effect against *P. aeruginosa.* Further investigation is required to pinpoint and to fully understand the effect of micafungin on the TCA cycle. Further investigations of its effects (at this or higher concentration) on the pseudomonal intracellular metabolic profile and the pseudomonal proteomic profile might pinpoint micafungin's most possible mechanism of inhibition and might facilitate the future selection of agents that interfere with biofilm architecture and development. Additionally, this work showed the importance of metabolomics as a new advanced analytical tool to investigate altered biochemical pathways in *P. aeruginosa*.

## Supplementary Information

**Additional file 1: Figure. S1. **Permutation tests for the statistical validation of the OPLS-DA models generated from *P. aeruginosa *samples treated with **(A)** micafungin 100 μg/mL and **(B)** azithromycin, 8 μg/mL compared to control. A permutation test was performed with 100 random permutations. Predictability Q2 (blue squares) and variability R2Y (green circles) values from the permuted analysis (bottom left) are lower than those of the initially generated model (top right).

## Data Availability

Data will be made available on request.

## Abbreviations

AHL: Acyl-homoserine lactone molecules
AI: Autoinducers
*C. albicans*: *Candida albicans*
CLSI: Clinical and Laboratory Standards Institute
CLSM: Confocal laser scanning microscopy
ddMS/MS: Data-dependent MS/MS
eDNA: Extracellular nucleic acids
EPS: Extracellular polymeric substance
ESI-: Negative electrospray ionization
ESI +: Positive electrospray ionization
FDR: False discovery rate
LC–MS: Liquid chromatography-mass spectrometry
MIC: Minimum inhibitory concentration
MM: Minimal media
MSI: Metabolomics standards initiative
NB: Nutrient broth
OPLS-DA: Orthogonal partial least square discriminative analysis
*P. aeruginosa*: *Pseudomonas aeruginosa*
PCA: Principal component analysis
PLS-DA: Partial least square discriminative analysis
QS: Quorum sensing
QSI: Quorum sensing inhibitor
RT: Retention time
SD: Standard deviation
VIP: Variable importance in the projection
