# Supplementary material for: Micafungin effect on Pseudomonas aeruginosa metabolome, virulence and biofilm: potential quorum sensing inhibitor
Source: AMB Express. 2023 Feb 20;13:20. doi: 10.1186/s13568-023-01523-0 (PMC9941417; doi:10.1186/s13568-023-01523-0)
Supplement: Supplementary file 1 — Additional file 1: Figure. S1. Permutation tests for the statistical validation of the OPLS-DA models generated from P. aeruginosa samples treated with (A) micafungin 100 μg/mL and (B) azithromycin, 8 μg/mL compared to control. A permutation test was performed with 100 random permutations. Predictability Q2 (blue squares) and variability R2Y (green circles) values from the permuted analysis (bottom left) are lower than those of the initially generated model (top right). [file 13568_2023_1523_MOESM1_ESM.pdf]

AMB Express

Metabolomic analysis of Micafungin as a Potential Quorum Sensing Inhibitor against *Pseudomonas aeruginosa*

Duaa M. Hijazi[1], Lina A. Dahabiyeh[1,*], Salah Abdelrazig[2,3], Dana A. Alqudah[4], Amal G. Al-Bakri[5,*]

[1]Department of Pharmaceutical Sciences, School of Pharmacy, The University of Jordan, Amman, 11942, Jordan.
[2]Department of Pharmaceutical Chemistry, Faculty of Pharmacy, University of Khartoum, P.O. Box 1996, Khartoum 11115, Sudan.
[3]Centre for Analytical Bioscience, Advanced Materials and Healthcare Technologies Division, School of Pharmacy, University of Nottingham, Nottingham NG7 2RD, U.K.
ORCID: orcid.org/0000-0001-6231-1267.
[4]Cell Therapy Center, The University of Jordan, Amman 11942, Jordan.
[5]Department of Pharmaceutics and Pharmaceutical Technology, School of Pharmacy, The University of Jordan, Amman, 11942, Jordan.

Corresponding authors:

Amal G. Al-Bakri
Department of Pharmaceutics and Pharmaceutical Technology, School of Pharmacy, The University of Jordan, Amman, 11942, Jordan

Email: agbakri@ju.edu.jo

Tel: +962-6 5355000 ext 23330

Fax: +962-6 5300250

ORCID: orcid.org/0000-0002-6299-5301

Lina A. Dahabiyeh
Department of Pharmaceutical Sciences, School of Pharmacy, The University of Jordan, Amman, 11942, Jordan
Email: l.dahabiyeh@ju.edu.jo
Tel: +962-6 5355000 ext 23306

Fax: +962-6 5300250

ORCID: orcid.org/0000-0002-4688-7052

(A)

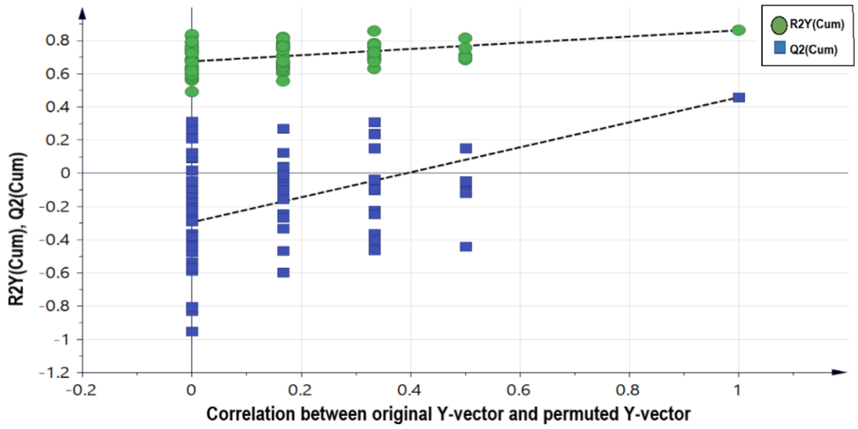

(B)

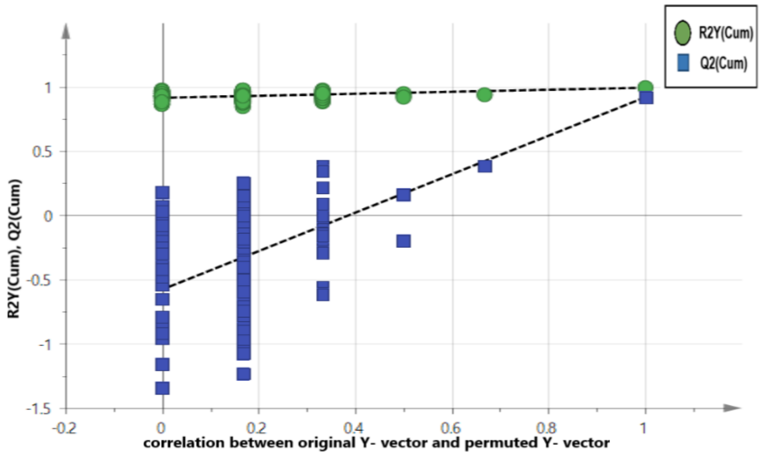

Figure. S1 Permutation tests for the statistical validation of the OPLS-DA models generated from *P. aeruginosa* samples treated with **(A)** micafungin 100 µg/mL and **(B)** azithromycin, 8 µg/mL compared to control. A permutation test was performed with 100 random permutations. Predictability Q^2 (blue squares) and variability R^2Y (green circles) values from the permuted analysis (bottom left) are lower than those of the initially generated model (top right)
